# Identification of hub genes and candidate herbal treatment in obesity through integrated bioinformatic analysis and reverse network pharmacology

**DOI:** 10.1038/s41598-022-22112-4

**Published:** 2022-10-12

**Authors:** Yuxing Tai, Hongying Tian, Xiaoqian Yang, Shixing Feng, Shaotao Chen, Chongwen Zhong, Tianjiao Gao, Xiaochao Gang, Mingjun Liu

**Affiliations:** 1 Changchun University of Chinese Medicine, Changchun, 130117 China; 2 Jilin Ginseng Academy, Changchun University of Chinese Medicine, Changchun, 130117 China; 3 Beijing University of Chinese Medicine, Beijing, 100029 China; 4 Department of Acupuncture and Tuina, Changchun University of Chinese Medicine, Changchun, 130117 China; 5 Acupuncture and Massage Center of the Third Affiliated Clinical Hospital, Changchun University of Chinese Medicine, Changchun, 130117 China

**Keywords:** Genetics, Biomarkers, Diseases, Endocrinology, Medical research, Mathematics and computing

## Abstract

Obesity is a global epidemic elevating the risk of various metabolic disorders. As there is a lack of effective drugs to treat obesity, we combined bioinformatics and reverse network pharmacology in this study to identify effective herbs to treat obesity. We identified 1011 differentially expressed genes (DEGs) of adipose tissue after weight loss by analyzing five expression profiles (GSE103766, GSE35411, GSE112307, GSE43471, and GSE35710) from the Gene Expression Omnibus (GEO) database. We identified 27 hub genes from the protein–protein interaction (PPI) network by performing MCODE using the Search Tool for the Retrieval of Interacting Genes (STRING) database. Gene Ontology (GO) and Kyoto Encyclopedia of Genes and Genomes (KEGG) pathway enrichment analyses revealed that these hub genes have roles in the extracellular matrix–receptor interaction, cholesterol metabolism, PI3K-Akt signaling pathway, etc. Ten herbs (Aloe, Portulacae Herba, Mori Follum, Silybum Marianum, Phyllanthi Fructus, Pollen Typhae, Ginkgo Semen, Leonuri Herba, Eriobotryae Folium, and Litseae Fructus) targeting the nine hub genes (COL1A1, MMP2, MMP9, SPP1, DNMT3B, MMP7, CETP, COL1A2, and MUC1) using six ingredients were identified as the key herbs. Quercetin and (-)-epigallocatechin-3-gallate were determined to be the key ingredients. Lastly, Ingredients-Targets, Herbs-Ingredients-Targets, and Herbs-Taste-Meridian Tropism networks were constructed using Cytoscape to elucidate this complex relationship. This study could help identify promising therapeutic targets and drugs to treat obesity.

## Introduction

Obesity is a global health problem without a definitive cure. Being overweight enhances the risk of chronic diseases, including type 2 diabetes, cardiovascular disease, cancer, and reproductive disorders^1–4^. Patients with moderate or severe obesity will also face higher all-factor mortality^5^. A study revealed that the prevalence of high body mass index (BMI) has increased globally over recent decades. To date, no country has been able to reduce its obesity epidemic by following evidence-based policies^6–8^. Although dietary and lifestyle modifications are considered primary solutions to treat obesity, recent studies recommend medications combined with lifestyle modifications to reduce weight among patients having BMI ≥ 27 kg/m^2^ and other associated diseases, or BMI ≥ 30 kg/m^29^. Most drugs used to manage obesity focus on appetite control. These drugs help in reducing food intake by stimulating pro-opiomelanocortin (POMC) neurons to promote satiation, ultimately leading to weight loss^10^. However, these current anti-obesity drugs are associated with side effects such as insomnia, dry mouth, constipation, adverse gastrointestinal reactions, and acute liver injury ^11,12^. Therefore, effective alternative strategies are urgently required.

Several studies focus on using Chinese herbs and other natural medications to treat obesity^13^ owing to the advantages of traditional Chinese medicine (TCM) in enhancing drug safety and reducing complications due to the homologous characteristics of medicine and food^14^. A systematic review shows that herbal medicines can effectively reduce weight, BMI, waist circumference, hip circumference, and body fat^15^. They exert anti-obesity effects by suppressing appetite and reducing energy intake, inhibiting pancreatic lipase activity and fat absorption, stimulating thermogenesis and energy expenditure, increasing lipolysis, and reducing lipogenesis^15^. Moreover, a randomized controlled trial showed that safflower oil intake could modify body weight and shape without lifestyle adjustments and also regulate blood pressure and insulin resistance compared with the placebo^16^. Drug discovery is associated with challenges such as long development cycles, high costs and turnover rates, and constantly changing regulatory requirements, which are not ideal for inventors^17^. As opposed to developing brand-new drugs, the extraction of natural ingredients from known herbs has various advantages including considerable saving of both time and economic costs. Therefore, it is necessary to identify novel and natural compounds that are suitable for the treatment of obesity.

Owing to the popularity of gene-microarray and RNA sequencing, integrated bioinformatics has been widely used to analyze high-throughput sequencing data of various diseases. By identifying differentially expressed genes (DEGs) and screening suitable hub genes, disease-related signaling pathways and their mechanisms can be analyzed, becoming the basis for predicting targeted drugs. In addition, network pharmacology has attracted considerable attention for the further exploration of the relationship between drugs, diseases, and targets by using various databases for analysis and simulation^18^. Many studies have clarified the targets and mechanisms of several Chinese medicines and their components in treating diseases using this technology. However, only a few have focused on mining new targeted drugs. In this study, we used an alternative strategy and reversed this process. Accordingly, the Gene Expression Omnibus (GEO, https://www.ncbi.nlm.nih.gov/geo/) database and the Traditional Chinese Medicine Systems Pharmacology Database and Analysis Platform (TCMSP, https://tcmspw.com/tcmsp.php) were used to identify the hub genes associated with obesity, explore the relevant mechanism through enrichment analysis, and predict the herbs and their ingredients with potential anti-obesity effect.

## Results

### Identification of DEGs after weight loss

**Figure 1:**
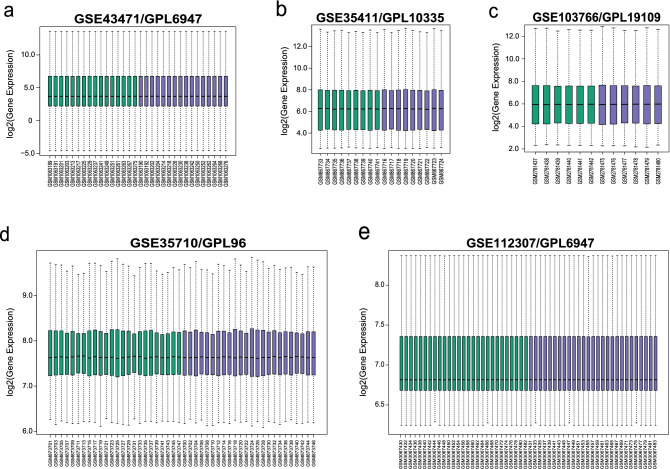
Box-plots of the expression profiles after consolidation and standardization. The x-axis label represents the sample symbol and the y-axis label represents gene expression values. The black line in the box-plot represents the median value of gene expression. (**a**) Standardization of GSE43471, (**b**) Standardization of GSE35411, (**c**) Standardization of GSE103766, (**d**) Standardization of GSE35710, (**e**) Standardization of GSE112307.

**Figure 2:**
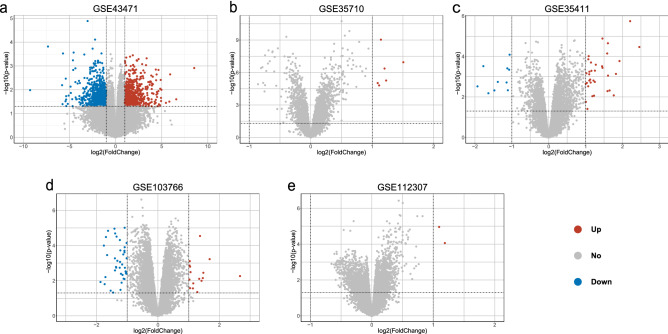
Volcano plot to identify differentially expressed genes (DEGs). (**a**) GSE43471, (**b**) GSE35710, (**c**) GSE35411, (**d**) GSE103766, (**e**) GSE112307. The x-axis label represents fold changes and the y-axis label represents the *p*-values. Red dots represent the 498 upregulated genes and green dots represent the 513 downregulated genes.

After standardizing gene sets (Fig. 1), 1011 DEGs (|logFC|> 1, *p* < 0.05) were screened out from GSE103766, GSE35411, GSE112307, GSE43471, and GSE35710 based on the above method. The results included 513 downregulated and 498 upregulated genes, as shown in the volcano plot (Fig. 2 and Supplementary Table S1). The abscissa in the volcano plot is log2 (fold change) value, and the ordinate is log10 (*p*-value).

### PPI network analysis and identification of hub genes

**Figure 3:**
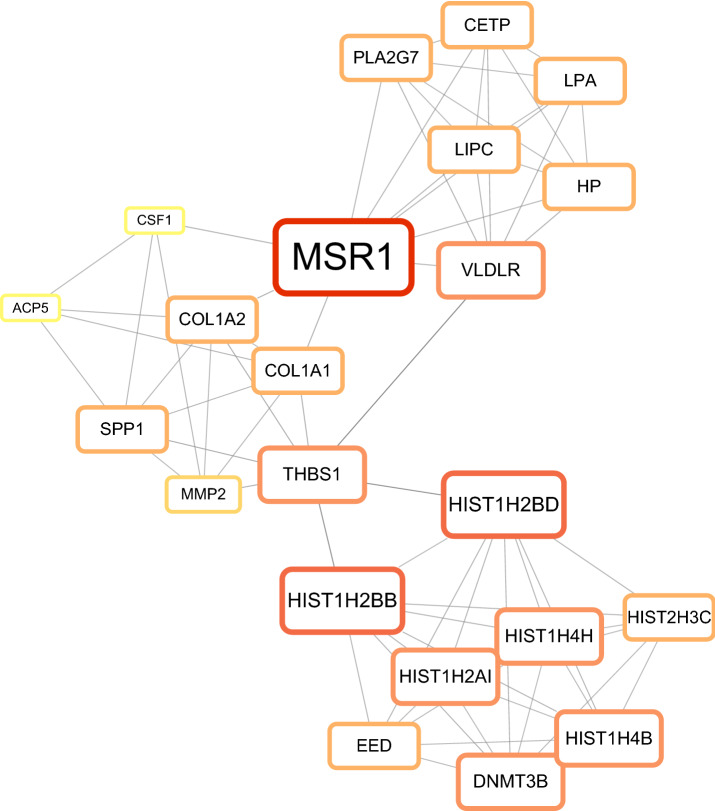
Subnetwork of 27 hub genes from the protein–protein interaction (PPI) network. Node size and temperature color reflect the degree of connectivity (bigger node represents a higher degree and smaller node represents a lower degree; red node represents a higher degree and yellow node represents a lower degree).

As shown in Supplementary Fig. S1, the PPI network of DEGs, based on the Search Tool for the Retrieval of Interacting Genes (STRING) database, includes 584 nodes and 1417 edges. Using the MCODE plugin in Cytoscape software, the most significant modules (score = 6.667) were recognized from the PPI network as comprising 27 hub genes, including ACP5, CETP, COL1A1, COL1A2, CSF1, DNMT3B, EED, HIST1H2AI, HIST1H2BB, HIST1H2BD, HIST1H4B, HIST1H4H, HIST2H3C, HP, LCN2, LIPC, LPA, MMP2, MMP7, MMP9, MSR1, MUC1, PLA2G7, SPP1, THBS1, THBS2, and VLDLR (Table 1 and Fig. 3).

**Table 1:** The MCODE scores of hub-genes.

Gene symbol	MCODE score
HIST1H4B	5.785714286
MMP2	5.474358974
HIST1H2BB	5.785714286
MMP9	5
HIST1H4H	5.785714286
CETP	6
HP	6
CSF1	5.571428571
HIST1H2AI	5.785714286
MUC1	5
COL1A2	6.611111111
HIST1H2BD	5.785714286
THBS2	5.2
EED	6
THBS1	6.611111111
COL1A1	5.981818182
ACP5	6
LCN2	5
DNMT3B	5.785714286
MSR1	6
LPA	6
LIPC	6
PLA2G7	6
SPP1	5.621212121
MMP7	5.066666667
VLDLR	6
HIST2H3C	6

### GO enrichment analysis of hub genes

**Figure 4:**
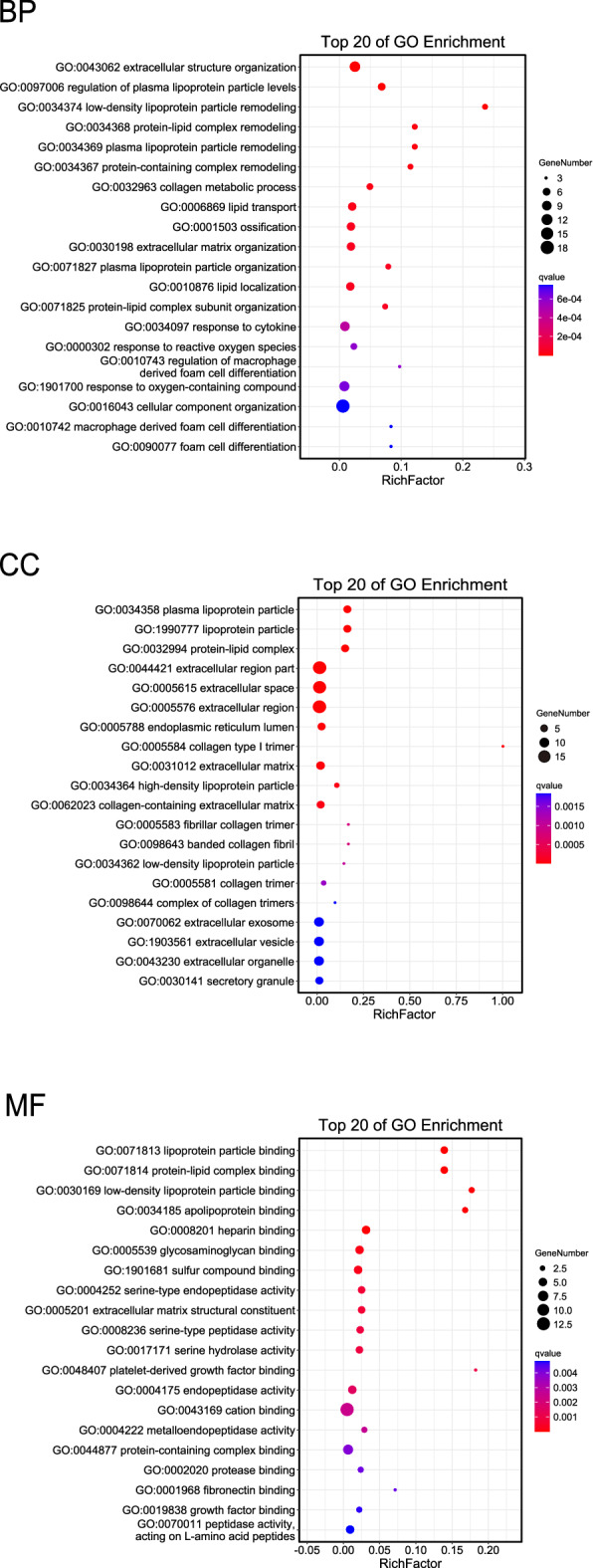
Biological functions based on Gene Ontology (GO) analysis of obesity-related hub genes. Advanced bubble chart shows significance in GO enrichment items of hub genes in three functional groups: biological process (BP), cell composition (CC), and molecular function (MF). The x-axis label represents the gene ratio (Rich Factor) and the y-axis label represents GO terms.

An enrichment analysis bubble chart was drawn under GO level 2 classifications using Omicshare tools (Fig. 4 and Supplementary Table S2). As shown in the figure, hub genes were significantly enriched in regulating plasma lipoprotein particle levels, lipid transport, extracellular matrix (ECM) organization, response to reactive oxygen species, and the oxygen-containing compound for biological process (BP). The hub genes were significantly enriched for cell composition (CC) in lipoprotein particles, extracellular regions, ECM, extracellular exosomes, and secretory granules. For molecular function (MF), the hub genes were significantly elevated in lipoprotein particle binding, glycosaminoglycan binding, ECM structural constituents, and peptidase activity.

### KEGG pathway enrichment analysis of hub genes

**Figure 5:**
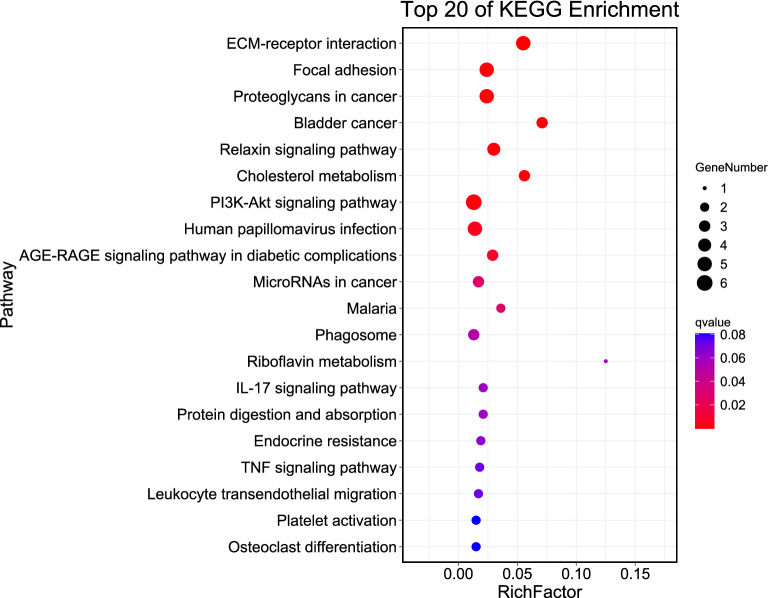
Kyoto Encyclopedia of Genes and Genomes (KEGG) pathway analysis of hub genes. The x-axis label represents the gene ratio (Rich factor) and the y-axis label represents the pathway.

KEGG pathway enrichment analysis showed that the hub genes were primarily enriched in ECM–receptor interaction, cholesterol metabolism, PI3K-Akt, IL-17, and TNF signaling pathways, endocrine resistance, and leukocyte transendothelial migration (Fig. 5 and Supplementary Table S3).

### Screening of active ingredients

We converted 27 gene names of the hub genes into protein names that could be recognized through the TCMSP database using the Universal Protein Resource (Uniprot). Moreover, the hub genes can be input in the required format to identify potential herbs with anti-obesity effects from the TCMSP database. After excluding the genes that were not present in the databases or those that had no related ingredients, nine were screened for further research, namely, COL1A1, MMP2, MMP9, SPP1, DNMT3B, MMP7, CETP, COL1A2, and MUC1. These genes corresponded to 16 ingredients [(-)-epigallocatechin-3-gallate (EGCG), arachidonic acid, arctiin, baicalein, beta-carotene, capillarisin, deoxypodophyllotoxin, ellagic acid, fisetin, irisolidone, luteolin, matrine, nobiletin, quercetin, rutaecarpine, tanshinone IIa] showing adequate OB and DL values (OB ≥ 30%, DL ≥ 0.18) (Supplementary Table S4).

### Screening and annotation of key herbs

There were 254 herbs with active ingredients in the databases. The top 10 herbs were Aloe, Portulacae Herba, Mori Follum, Silybum Marianum, Phyllanthi Fructus, Pollen Typhae, Ginkgo Semen, Leonuri Herba, Eriobotryae Folium, and Litseae Fructus. These were associated with more DEGs (related genes = 6) and were, therefore, selected as crucial herbs in our study and annotated using Chinese pharmaceutical properties (CMPs), including characters, tastes, and meridian tropisms (Table 2).

**Table 2:** The CMPs of key hub genes.

Herbs	Characters	Tastes	Meridian tropisms
Aloe	Cold	Bitter	Liver, stomach, large intestine
Portulacae herba	Cold	Acid	Liver, large intestine
Mori follum	Cold	Sweet, bitter	Lung, liver
Silybum marianum	Cool	Bitter	Liver, gallbladder
Phyllanthi fructus	Cool	Sweet, acid, astringent	Lung, stomach
Pollen typhae	Calm	Sweet	Liver, pericardium
Ginkgo semen	Calm	Sweet, bitter, astringent	Lung, kidney
Leonuri herba	Cold	Botter, symplectic	Liver, pericardium, bladder
Eriobotryae folium	Cold	Bitter	Lung, stomach
Litseae fructus	Warm	Symplectic	Spleen, stomach, kidney, bladder

### Construction of ingredients-targets, herbs-ingredients-targets, and herbs-taste-meridian tropism networks

**Figure 6:**
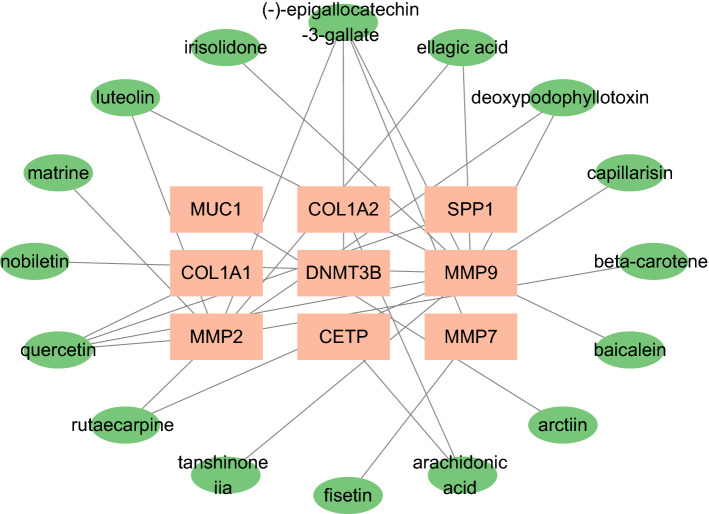
Ingredients-Targets network. Nine orange nodes represent the target genes, whereas the 16 green nodes represent the active compounds. The edges represent the interaction between the compounds and targets.

We screened the key ingredients in treating obesity using an Ingredients-Targets network containing 25 nodes and 27 edges (Fig. 6). The nine orange nodes represent the target genes and 16 green nodes represent the active ingredients. As most genes could be linked (degree = 4), quercetin and EGCG were considered the most critical components in the treatment of obesity.

**Figure 7:**
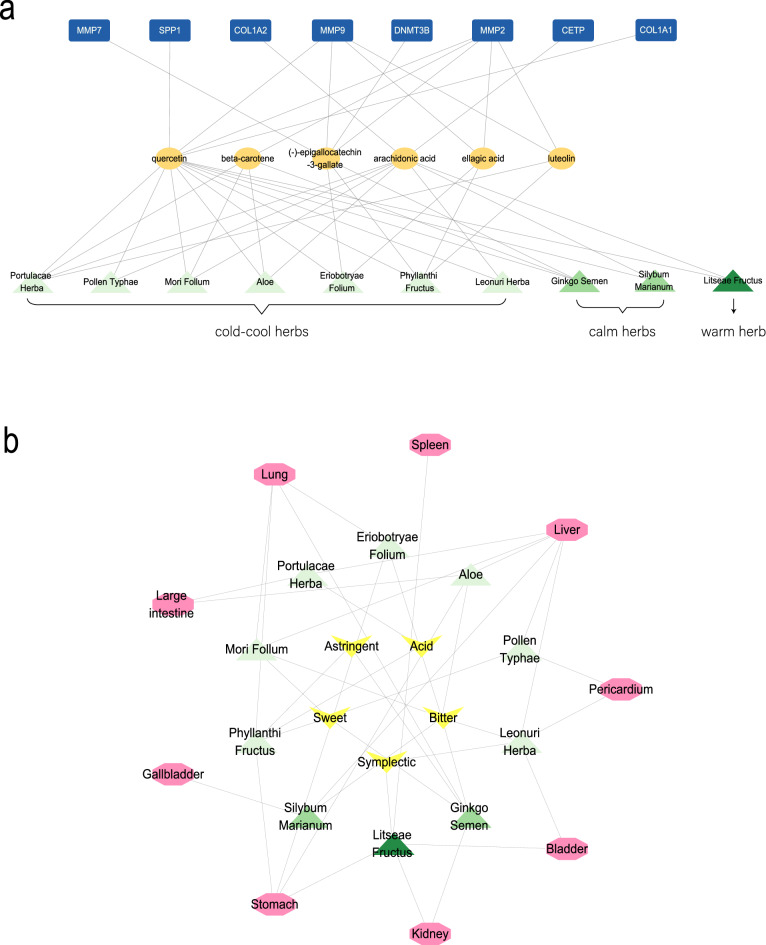
Herbs-Ingredients-Targets network (**a**) and Herbs-Taste-Meridian tropism (**b**) network. (**a**) Yellow nodes represent the active ingredients and the blue nodes represent the target genes. (**b**) Yellow nodes represent tastes and purple nodes represent meridian tropisms. In all networks, the light green nodes represent cold-cool herbs, medium green nodes represent calm herbs, and dark green nodes represent warm herbs.

As shown in Fig. 7a, the Herbs-Ingredients-Targets network containing 24 nodes and 43 edges was constructed to demonstrate the relationship between them: the 10 green nodes represent the key herbs and the six yellow nodes represent the active ingredients in them; the eight blue nodes depict the target genes. By analyzing the network, Phyllanthi Fructus and Portulacae Herba were associated with the most ingredients (degree = 4). Moreover, quercetin was the most frequent active ingredient (degree = 23) found in all herbs. Regarding gene targets, MMP2 was targeted by most ingredients (degree = 5) followed by MMP9 (degree = 4). Other genes were only acted upon by one component (degree = 1).

We also established the Herbs-Taste-Meridian tropism network containing 24 nodes and 40 edges to clarify the distribution of CMPs (Fig. 7b). Five yellow nodes represent tastes and eight purple nodes represent meridian tropisms. To indicate different characters, we presented 10 nodes of herbs having different greens (light green, medium green, and dark green). Regarding characters, cold-cool herbs like Mori Follum were the most frequent (nodes = 7), followed by herbs having calm (nodes = 2) and warm (nodes = 1) characters. In terms of taste, herbs were mostly bitter (edges = 6), followed by sweet (edges = 4), acid (edges = 2), symplectic (edges = 2), and astringent (edges = 2). Regarding meridian tropism, most herbs belonged to the liver meridian (edges = 6), followed by the stomach and lung (edges = 4), large intestine (edges = 2), bladder (edges = 2), kidney (edges = 2), pericardium (edges = 2), spleen (edges = 1), and gallbladder (edges = 1) meridians.

## Discussion

Obesity causes energy imbalance due to excess energy intake (intake > expenditure). Controlling energy intake and expenditure is the key to maintaining metabolism and weight loss^12^. Most anti-obesity drugs lead to weight loss by promoting satiety and reducing food intake and calorie absorption. The TCM theory has different views. Although there is no objection with respect to the influence of energy balance on body weight depending on the law of conservation of energy, TCM theory expounds on obesity from the perspective of body functions other than the dynamic equilibrium of energy and nutrition. Based on TCM, obesity, as a physical state, results from the combined influence of various congenital constitutions and living habits. The obese population can be divided into TCM syndrome types based on their body function status rather than the degree of obesity depending on BMI, abdominal circumference, and other data^17^. The TCM theory consistently implements this holistic dialectical thought to discuss several biomedical problems and provides new treatments for various diseases^18^. Owing to the side effects of the current drugs used for weight loss on one hand and the safety of natural components on the other, it is important to predict and further explore potential TCM-based therapies.

Heredity is an important factor contributing to obesity. About 40%–70% of BMI differences among individuals are attributed to genetic differences^19^. Data from genome-wide association studies indicate more than 140 genetic chromosomal regions associated with obesity, indicating genetic involvement^20^. About 60% of the cases of obesity are due to genetic differences^21^. Claussnitzer et al.^22^ observed that regulating the expression of related genes in adipose tissue could reduce weight and enhance energy consumption in mice without altering their physical activity or appetite. The mechanism is associated with inhibiting adipocyte precursor mitochondria thermogenesis via the obesity-related FTO allele in a tissue-autonomous way. A meta-analysis^19^ revealed that 97 genetic loci associated with BMI comprised less than 3% (about 2.7%) of BMI variation. In contrast, 21% of BMI variation was likely due to common genetic variation, indicating that any single gene could not explicitly explain BMI variation. Microarray analysis in systems biology is an effective tool to explore the potential genes and pathways in different diseases. It is performed by detecting tens of thousands of gene expression information using a high-throughput pattern^23^. The GEO database is the most comprehensive public functional genomics database that includes gene expression, mutation, modification, and other related information^24^. Currently, the GEO database has 65,775 microarray expression profiling by array that has been queried and downloaded by users for free. Therefore, this study was conducted based on the appropriate microarray data from the GEO database. Additionally, the expression data of the same population before and after weight loss was selected from this database in the current study, rather than data from individuals who were obese or of normal weight, to avoid the influence of individual genetic factors during the acquisition and adjustment of obesity.

GO and KEGG enrichment analysis of hub genes revealed that most genes were enriched in ECM-reception interaction, including six DEGs. The ECM is a complex structure with different proteins, proteoglycans, and polysaccharides. It provides a scaffold for cells to control biological processes such as cell adhesion, migration, repair, survival, and development. Obesity is characterized by the extensive expansion of adipose tissue. Thus, ECM remodeling and recombination provide adequate space for adipocyte proliferation and the formation of new fat cells by the adipogenesis of precursor cells^25^. In addition, this process forms new blood vessels, which are essential for the expansion of healthy adipose tissue. In addition, adipose tissues require a vascular network to receive oxygen and nutrients^26^. Failure of this process will lead to adipocyte necrosis and hypoxia, which can cause chronic inflammation and fibrosis and induce other chronic diseases. Therefore, the growth and expansion of adipose tissue depend on angiogenesis. Intervention with angiogenesis inhibitors among obesity models can reduce the number of blood vessels in adipose tissues^27^ and reduce weight without impacting food intake^28^.

In this study, we constructed an Ingredients-Targets network using Cytoscape. Next, we screened out two compounds as crucial ingredients in treating obesity (quercetin and EGCG), which could each target four hub genes considered key in treating obesity. Quercetin is a plant-derived flavonoid with anti-inflammatory and anti-obesity effects^29^. Related studies indicate that quercetin also has antioxidant and cardioprotective effects. Its anti-obesity effect is due to the inhibition of adipogenesis by inhibiting the gene expression of peroxisome proliferator-activated receptor-γ (PPAR-γ)^30^. Quercetin is reported to significantly alleviate high-fat-diet–induced obesity and influence the production of metabolites linked to obesity-related inflammation and oxidative stress by regulating gut microbiota and metabolites^31^. EGCG is a bioactive polyphenol with antioxidant and anti-inflammatory effects^32^. Treatment with EGCG leads to reduced visceral adiposity and loss in body weight and is associated with the regulation of Beclin1-dependent autophagy in white adipose tissue^33^. Moreover, EGCG can inhibit adipocyte differentiation and maturation via the PI3K-AKT-FOXO1 pathway^34^.

We identified 10 key herbs in treating obesity, many of which have been widely used clinically for weight loss. Eriobotryae Folium can alleviate visceral and central obesity, insulin resistance, dyslipidemia, oxidative stress, and inflammation of metabolic syndrome in high-fat-diet models^35^. Aloe can inhibit obesity-induced inflammation by activating AMPK expression in muscle and reducing proinflammatory cytokines in white adipose tissue^36^. Silybum Marianum has lipid-lowering, antihypertensive, antidiabetic, anti-atherosclerotic, hepatoprotective, and anti-obesity effects and is known for its antioxidant, anti-inflammatory, and β-cell regenerative effect; enhancement of insulin sensitivity; and inhibition of gluconeogenesis and Glut4-mediated transport^37^. However, further investigations, such as randomized controlled trials, are required to verify the clinical effects and elucidate its anti-obesity mechanisms.

One of the innovations of this study was the construction of the Herbs-Taste-Meridian Tropism network and understanding the CMP rules of herbs with promising effects. Although several scholars have begun to study the weight-loss effect of Chinese herbs, research on potential herbs based on CMP is relatively insufficient. CMP is an integral aspect of the TCM theory^38^ that has guided the clinical practice of TCM practitioners for thousands of years as the primary prescription basis. Some research groups have studied Hedysarum Multijugum Maxim, Coicis Semen, and other Chinese herbs based on the theories of Yin-Yang and Chinese herbal characters. They found that warm-hot herbs could increase cAMP, prostaglandin E2, and other heat-related metabolites in rats with elevated body temperature. Cold-cool herbs could elevate metabolites such as histidine, tyrosine, lipid, and inositol and exert a cooling effect^39^. A study indicated that^40^ the characters, tastes, and meridian tropisms of Chinese herbs correlate with human tissues and organs, depicting a nonlinear relationship. Warm herbs have evident effects on the upper part of the liver, heart, and spinal cord, whereas hot herbs affect the stomach, kidneys, and small intestine. According to the theory of TCM, Schisandrae Chinensis Fructus tastes like acid, and acid herbs correspond to the liver. Therefore, Schisandrae Chinensis Fructus is traditionally used in a clinical setting as a liver tonic. Recent studies have revealed that it can alleviate the symptoms of metabolic syndrome by inhibiting oleic acid–induced fatty liver^41^. From the perspective of meridian tropism, Sanjiao Meridian has a significant impact on the thymus, brain, upper spine, and medulla. In contrast, the Pericardial Meridian has significant effects on the stomach, kidneys, small intestine, and medulla. The above studies could not completely summarize the relationship between CMP and disease targets and the therapeutic mechanism. However, they did explain its regularity and objectivity to a certain extent.

### Limitations

Discrepancies in gender, body weight, age, treatments, platforms, and other factors may have influenced the identification of hub genes, as we used five distinct datasets from the GEO database. Thus, further studies on these genes are required for validation. Next, the two key ingredients and 10 key herbs predicted in this study should be verified using clinical and animal experiments to confirm their effectiveness and heterogeneity in obesity therapy. Lastly, further studies are required to validate the association between drug properties and CMPs and to elucidate potential mechanisms of action.

## Conclusions

In this study, we identified 27 hub genes associated with weight loss and analyzed their related signaling pathways using publicly available databases. Additionally, we predicted several potential natural compounds and Chinese herbs targeting these genes using the TCMSP platform. Quercetin and EGCG were screened out as crucial ingredients. Aloe, Portulacae Herba, Mori Follum, Silybum Marianum, Phyllanthi Fructus, Pollen Typhae, Ginkgo Semen, Leonuri Herba, Eriobotryae Folium, and Litseae Fructus were screened out as key herbs. These herbs and compounds are expected to be effective in treating obesity. Supplementary investigations involving randomized controlled trials and molecular biology studies are warranted to verify these findings.

## Methods

### Downloading and normalization of microarray data

The search strategy included (“obesity”[MeSH terms] OR obesity [All Fields]) AND “Homo sapiens”[porgn] AND “Expression profiling by array”[Filter]. The inclusion criteria were as follows: (i) subcutaneous adipose tissue (SAT) from patients who were obese after lifestyle-based weight-loss interventions; (ii) SAT of patients before weight-loss interventions were considered as controls.

Based on the above retrieval strategy, the five gene expression profiles that were selected for analysis from the GEO database were GSE103766^42^, GSE35411^43^, GSE112307^44^, GSE43471^45^, and GSE35710^46^, and their platforms were GPL19109, GPL10335, GPL6947, GPL6947 (same as previous), and GPL96. After the five data sets were retrieved and standardized using the GEOquery^47^ package in R software (version 4.1.3)^48^, ggplot2 package was used to design box-plots for gene expression data^49^.

### Identification of differentially expressed genes (DEGs)

DEGs with a threshold criterion of |log FC |> 1 and *p*-value < 0.05^50^ in SAT samples were screened using the linear models for microarray data package after incorporating weight-loss intervention^51^. Pheat map, ggplot2, and RColorBrewer packages^52,53^ were used to formulate the volcano plots of the DEGs.

### Creation of the PPI network and identification of hub genes

After summarizing and removing duplicates DEGs, the PPI network of DEGs was determined to identify the most significant clusters of DEGs using STRING (version 11.5; https://cn.string-db.org/) having a combined score > 0.4 as the cutoff point. After STRING analysis, Cytoscape (version 3.9.1), an open-source bioinformatics software platform used for network analysis to visualize their associations, was used to visualize and identify the PPI network. MCODE plugin (version 2.0.0) was used to identify the hub genes, and the parameters of DEG clustering and scoring were as follows: MCODE score = 6.667, Degree Cut-off = 2, Node Score Cutoff = 0.2, k-score = 2, and Max. Depth = 100.

### GO and KEGG pathway enrichment analyses

GO enrichment analysis annotates the genetic information based on three aspects: MF, BP, and CC. It is widely used in mining biological function information and the corresponding biological mechanism in microarray results^54^. KEGG (https://www.genome.jp/kegg/) is a comprehensive database involving pathways, genes, compounds, drugs, and diseases for annotating the biological functions of genes and genomes at the molecular level^55^. GO and KEGG enrichment analyses were performed on the screened hub genes using Omicshare tools, an online platform for data analysis and gene annotation of gene DENOVO (https://www.omicshare.com/tools/).

### Screening of active ingredients

When retrieving drug information associated with specific genes (targets) from the TCMSP database, there are requirements on the format of input information. TCMSP can only identify protein names. Nevertheless, the gene expression matrices obtained from the previous steps had been written by the gene names. We needed to transform gene names into protein names that TCMSP could recognize to successfully compare the information of gene targets with Chinese herbs in TCMSP. This step was completed using UniProt (https://www.uniprot.org/help/about), a free comprehensive online database for protein sequences and their annotations^56^. After entering all protein names for the hub genes, we selected the active ingredients of Chinese herbs based on their oral bioavailability (OB) and drug-likeness (DL) values (OB ≥ 30%, DL ≥ 0.18).

### Screening and annotation of key herbs

After screening the herbs containing active ingredients that could treat obesity, the relationship between the herbs and target genes was studied. If different components in a particular herb were associated with the same target, only one association was considered between the herb and the target gene. Herbs associated with more target genes were considered more significant in treating obesity. We defined a part of the herbs acting on more target genes as the key herbs in treating obesity. Then, referring to the Chinese Pharmacopoeia 2020 Edition (I), key herbs were annotated with CMPs, such as characters, tastes, and meridian tropisms.

### Construction of ingredients-targets, herbs-ingredients-targets, and herbs-taste-meridian tropism networks

We established the Ingredients-Targets, Herbs-Ingredients-Targets, and Herbs-Taste-Meridian Tropism networks using Cytoscape to determine the association between the above nodes. The importance of each node in the network was determined based on the degree value of the network topology parameter. Thus, the importance of the node changed significantly with the increase in degree value, and the degree value was represented by the edge connecting the node^57^.

## Supplementary Information

Supplementary Information 1.

Supplementary Information 2.

Supplementary Information 3.

Supplementary Information 4.

Supplementary Information 5.

## Data Availability

The data that support the findings of this study are available in GEO database (http://www.ncbi.nlm.nih.gov/geo), reference number [GSE103766, GSE35411, GSE112307, GSE43471, and GSE35710].
