# Supplementary figures and images for: Identification of hub genes and candidate herbal treatment in obesity through integrated bioinformatic analysis and reverse network pharmacology
Source: Sci Rep. 2022 Oct 12;12:17113. doi: 10.1038/s41598-022-22112-4 (PMC9556576; doi:10.1038/s41598-022-22112-4)

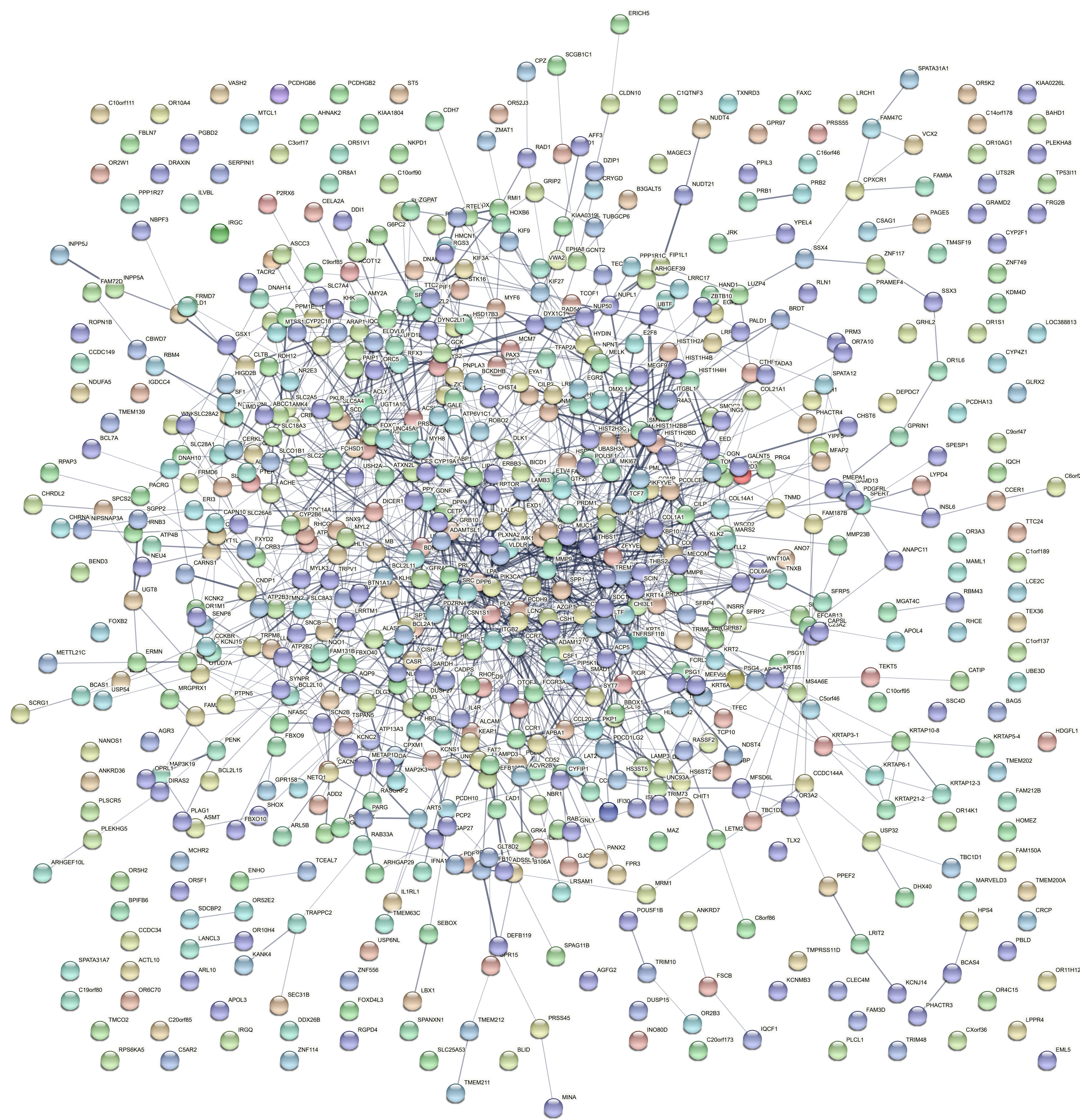

Supplement: Supplementary file 1 — Supplementary Information 1. [file 41598_2022_22112_MOESM1_ESM.pdf]
